# Supplementary material for: Prevalence of tissue transglutaminase antibodies and IgA deficiency are not increased in juvenile idiopathic arthritis: a case-control study
Source: Pediatr Rheumatol Online J. 2023 Oct 5;21:110. doi: 10.1186/s12969-023-00890-z (PMC10557180; doi:10.1186/s12969-023-00890-z)
Supplement: Supplementary file 1 — Supplementary Material 1 [file 12969_2023_890_MOESM1_ESM.docx]

Prevalence of tissue Transglutaminase Antibodies and IgA deficiency are not increased in Juvenile Idiopathic Arthritis: A case-control study.

Angela Taneja Kohli^1,2^, Aimee O. Hersh^3^, Lori Ponder^2^, Lai Hin Kimi Chan^1^, Kelly A. Rouster-Stevens^1,2^, Anne E Tebo^4^, Subra Kugathasan^1,2^, Stephen L. Guthery^3^, John F. Bohnsack^3^, Sampath Prahalad^1,2^

^1^Department of Pediatrics, Emory University School of Medicine, Atlanta, GA,

^2^Children’s Healthcare of Atlanta, Atlanta, GA,

^3^Department of Pediatrics, Spencer F. Eccles School of Medicine, University of Utah, Salt Lake City, UT

^4^University of Utah, Salt Lake City, UT,

Funding: Dr. Prahalad is supported in part by a grant from the Marcus Foundation Inc., Atlanta.

This work has been previously presented as a poster in the Pediatric Rheumatology Symposium in Houston, TX in 2017.

Correspondence to: Angela.Taneja@emory.edu

**ABSTRACT:**

**Background:** The prevalence of Celiac Disease (CD) in Juvenile Idiopathic Arthritis (JIA) has been reported to be 0.1% to 7% in various small studies. As a result of the limited number of research and their inconclusive results there are no clear recommendations for routine CD screening in asymptomatic patients with JIA. Our aim is to estimate the prevalence of IgA deficiency and tissue transglutaminase (tTG) IgA in a cohort of JIA followed in two large academic medical centers.

**Methods:** Serum was collected and stored from all subjects and analyzed in a reference laboratory for total IgA (Quantitative Nephelometry) and tTG IgA antibody levels (Semi-Quantitative Enzyme-Linked Immunosorbent Assay). Fisher’s exact tests were performed for statistical significance. Risk estimates (odds ratios) with 95% confidence intervals were calculated.

**Results:** 808 JIA and 140 controls were analyzed. Majority were non-Hispanic whites (72% vs 68% p=0.309). A total of 1.2% of cases were IgA deficient compared to none of the controls (p=0.373). After excluding IgA deficient subjects, 2% of cases had tTG IgA ≥4u/mL compared to 3.6% of controls (p=0.216) (OR = 0.5; 95% C.I= 0.1-1.4); and 0.8% of cases had tTG IgA >10u/mL compared to 1.4% of controls (p=0.627) (OR=0.5; 95%C.I=0.1-2.9).

**Conclusions:** Using the largest JIA cohort to date to investigate prevalence of celiac antibodies, the prevalence of positive tTG IgA was 0.8% and of IgA deficiency was 1.2%. The results did not demonstrate a higher prevalence of abnormal tTG IgA in JIA. The study did not support the routine screening of asymptomatic JIA patients for Celiac Disease (CD).

**KEYWORDS:**

tissue Transglutaminase IgA

tTG IgA

IgA deficiency

celiac antibodies

celiac disease

Juvenile Idiopathic Arthritis

prevalence

children

healthy controls

**BACKGROUND:**

Juvenile idiopathic arthritis (JIA) is a heterogeneous collection of chronic autoimmune arthropathies of childhood with a prevalence of about 1 in 1000 children under age 16 (1). Investigation of the co-occurrence of autoimmune phenotypes have shown that JIA patients have an increased prevalence of type 1 diabetes (2, 3). Relatives of children with JIA also have higher rates of autoimmunity (4). The prevalence of Celiac Disease (CD) in JIA has been reported to be 0.1% to 7% in several small studies (5-12). The detection of CD -specific IgA autoantibodies against the enzyme tissue transglutaminase (tTG IgA) is an accurate screening tool to diagnose or identify patients at risk for CD (13). Given the relatively high prevalence of IgA deficiency in relation to other autoimmune diseases in patients with CD, evaluation of this deficiency and use of CD -specific IgA autoantibodies against the enzyme tissue transglutaminase (tTG IgA) is recommended by current North American Society for Pediatric Gastroenterology, Hepatology and Nutrition guidelines (14).

Celiac disease, defined as a sensitivity to gluten in wheat and related proteins found in barley and rye, occurs in genetically susceptible individuals and manifests as an immune-mediated enteropathy as defined by changes seen on intestinal histology (14). The prevalence of CD in persons older than 6 years of age and younger than 20 years has been estimated to be 1.2% for non-Hispanic whites, 0.2% for Hispanics, and 0.1% for non-Hispanic blacks (15). CD can remain asymptomatic for years, it might be undiagnosed, or be misdiagnosed as a different disorder. Treatment consists of lifelong exclusion of gluten from the diet. Since asymptomatic individuals with some autoimmune disorders and chromosomal abnormalities can have a higher prevalence of CD, screening is recommended for specific populations (14, 16).

Celiac disease frequently coexists with other conditions which can delay its diagnosis and the introduction of a gluten-free diet. JIA patients are reported to have an increased risk for co-existing CD, but data is mixed and inconclusive. Some children thought to have JIA may have occult CD with extra intestinal manifestations. A gluten-free diet might help alleviate joint symptoms in patients with CD (17). It has also been reported that enthesopathy is more frequent in untreated CD subjects with positive tTG antibodies, as compared to those treated with gluten free diet who have cleared the tTG titer (18).

As a result of the limited number of studies and their inconclusive results there are no clear recommendations for routine CD screening in asymptomatic patients with JIA (19).

The objective of the present study was to investigate the prevalence of IgA deficiency and tTG IgA antibodies in a large JIA cohort compared to healthy autoimmunity free controls.

**METHODS:**

A case control design was utilized which included subjects from two large academic medical centers from Atlanta/GA and Salt Lake City/UT. Informed consent was obtained from all cases and controls under protocols approved by institutional review boards at Emory University and University of Utah.

Clinical and demographic data collected included disease status, gender, race, ethnicity, ILAR category, age at the time of sample collection and age of onset of JIA for cases. For cases with positive tTG antibodies, medical records were reviewed for gastrointestinal symptoms and results of gastroenterology evaluation if applicable.serum was collected and stored from all subjects and analyzed at a single time in a reference laboratory for total IgA (Quantitative Nephelometry) and tTG IgA antibody levels (Semi-Quantitative Enzyme-Linked Immunosorbent Assay). Standard reference levels of IgA at <7 mg/dl for IgA deficiency and tTG IgA 0-3 U/mL as negative, and > 4 U/mL as positive were applied as per manufacturer’s recommendations. For calculations, one case and control who had tTG levels >100 U/mL were designated as 100 u/mL, and the 9 JIA cases with undetectable levels of IgA were designated as 0 mg/dL. Chi-Square tests or Fisher’s exact tests were performed for statistical significance. Only healthy controls (ages 3 to 30 at the time of collection) were included. Those with unknown age of collection and IgA deficiency were excluded from the tTG IgA analysis All controls completed a questionnaire for the presence of autoimmune disorders at the time of enrollment and were enrolled only if they reported no autoimmunity.

**RESULTS:**

There were 140 healthy controls and 808 cases with JIA for analysis. JIA ILAR category information and demographic features of cases and controls is included in **Table 1** and Table 3 respectively, and the distribution of IgA and tTG values among the controls and JIA categories are shown in **Figure 1**.

Cases were 808 children and adolescents (65.8% female) aged 3 to 21 years at the time of enrollment who met the classification criteria established by International League of Associations for Rheumatology (ILAR) (20). The mean age of onset of JIA was 7.4 years. The mean age at the time of collection of serum was 10.9 years. Most of the JIA cases were non-Hispanic whites (78%). Controls were 140 healthy individuals, (54.3% female) aged 3 to 30 years of age at enrollment, without a history of autoimmunity. Controls completed a questionnaire for presence of autoimmune disorders at the time of enrollment and were enrolled only if they reported no autoimmunity among the controls or their first-degree relatives. Controls have been previously described (21). The mean age of the controls at the time of collection of serum was 20.7 years. Most of the healthycontrols were non-Hispanic whites (68%). Further demographic information is available in **Table 1**.

The mean level of IgA was 252 mg/dL among the 140 controls (Range 9-695 mg/dLand 186 mg/dL among the JIA cases (Range 0-754 mg/dL). There were 9 cases with JIA (1.2%) that had levels of IgA below the range for normal compared to none of the controls
(p = 0.373). The mean (median) level of tTG was 1.76 (1.0) U/mL among the 140 controls (Range 0->100 U/mL). The mean (median) level of tTG among the JIA cases was 0.97 (0.0) U/mL (Range 0->100 U/mL). After excluding the 9 IgA deficient subjects, 15 cases (1.9%) had tTG IgA > 4.0 u/mL compared to 5 controls (3.6%), a difference which was not significant (p=0.20). There was also no difference in the proportion of subjects with a tTG IgA >10.0 u/mL (0.88% of cases vs 1.4% of controls; p 0.29). There were also no significant differences in the prevalence of abnormal tTG between the control cohort and any of the JIA categories (Table 1). Since our controls were older than cases, we also repeated the analysis after only including the 82 controls aged 3-21 (mean 18.0 and SD 2.9). Only 2 out of 82 had tTG IgA > 4.0 u/mL, which was not statistically significant from the prevalence among the cases (p =0.74).

There were 15 cases with JIA that had a positive tTG described in **Table 4**. Five of the cases had been referred to and had been evaluated by gastroenterology for abdominal symptoms. Three had normal endoscopy with biopsies. Two others were diagnosed with CD histologically. Of the remaining 10 children, 2 had additional tTG testing done as standard of care after the date of serum collection and were negative. The remainder of the cases had no documentation of GI symptoms.

Majority of cases and controls with abnormal tTG IgA and IgA deficient as described in demographic Table 3 are female and non-Hispanic whites.

Table 4 describes the phenotypes of the 15 children with high tTG IgA in our study. To our knowledge, only two were diagnosed with celiac disease; a 15 year old male with tTG IgA of 30 with diagnosis of Polyarticular JIA RF positive and a 17 year old female with Oligoarticular JIA, both non-Hispanic whites.

**DISCUSSION:**

This is the largest JIA cohort investigated for the presence of CD antibodies to date. The North American Society for Pediatric Gastroenterology Hepatology and Nutrition (NASPGHAN) has developed an algorithm on the evaluation of the asymptomatic child in at-risk groups (14). CD is 3 to 10-fold higher in asymptomatic individuals with other autoimmune diseases or chromosomal abnormalities for which screening is recommended.^4,5^ It occurs in children and adolescents with gastrointestinal symptoms, dermatitis herpetiformis, dental enamel defects, osteoporosis, short stature, delayed puberty and persistent iron deficiency anemia; and in asymptomatic individuals with type 1 Diabetes, Down syndrome, Turner syndrome, Williams syndrome, selective immunoglobulin IgA deficiency and first degree relatives of individuals with celiac disease. Notably, JIA is not included as a recommendation for CD screening. It is recommended that serological testing of asymptomatic children for CD who belong to high-risk groups begin by 3 years of age provided they have had an adequate gluten-containing diet for at least one year before testing. It is also recommended that asymptomatic persons with negative serological tests who belong to groups at risk be considered for repeat testing at intervals. Although an intestinal biopsy is still considered necessary for confirmation of CD, serological tests are often used to identify individuals in whom the biopsy is indicated.

Recommended serological tests for CD include anti-deamidated gliadin peptide IgA/IgG (DGP IgA/DGP IgG), anti-endomysium IgA (EMA) and anti-tTG IgA antibodies; these laboratory tests are especially useful in individuals without gastrointestinal symptoms, those with conditions associated with CD, and for screening asymptomatic first-degree relatives of known cases (14). They have also been used in epidemiologic studies to determine the prevalence of CD. Due to variable and inferior accuracy the anti-gliadin antibodies (AGA) IgA/IgG, testing for these antibodies are no longer recommended (14). Tissue transglutaminase (tTG) IgA has high specificity for CD in individuals with normal IgA levels, and highly correlates with biopsy proven celiac disease. In identified persons post screening tTG IgA alone has high positive predictive values (median of 0.937) for biopsy proven celiac disease (22).

Selective IgA deficiency has a reported frequency of 1 in 39 in populations of both adult and childhood CD, compared to an estimated frequency of 1 in 600 in the general population (23). However, the absence of IgA anti-tTG antibodies in patients with IgA deficiency should be interpreted with caution, and IgG anti-tTG antibody levels or HLA typing should be measured to screen for CD.

We identified 9 prior reports investigating the prevalence of tTG in cohorts of JIA .The number of subjects with JIA ranged from 32-216 in these studies. Only 4 of the studies reported using healthy controls. While the prevalence of tTG positivity in these studies ranged from 0% to 53%, the median prevalence of 1.9% is similar to the results from our large cohort. In one study from Egypt reported by Gheita et al., (24) the prevalence of tTG positivity among 42 cases was 53%, but notably the prevalence among the 30 healthy controls was also high at 20%. Another study by Stoll et al., from the US reported tTG positivity of 20% among 10 controls compared to 14% among 42 children with JIA (25). The higher prevalence of tTG in the controls in these studies suggest these are likely outliers.

Since IgA deficiency can cause a false negative on a CD antibody test, it is recommended that serum IgA levels be measured when evaluating for CD. Of the studies reported in Table 4, only 3 specifically mentioned the prevalence of IgA deficiency in their cohorts which ranged from 0 to 0.9%. The prevalence of IgA deficiency was 0 among 108 Italian children with JIA (6) and 96 Turkish children with JIA (11).

While ours is the largest cohort, the study does have some limitations. Our controls were older compared to the cases which might explain in part why the controls had a higher prevalence of tTG positivity. Cases were enrolled from two different institutions. The study was performed on banked serum, so information regarding the diets of the subject in the year prior to testing was not recorded in the chart. To avoid false negative tests, we only included subjects older than 3 years of age. We did not test the 9 IgA deficient subjects for tTG or DGP IgG antibodies or for HLA (Human Leukocyte Antigen) antigens, and hence we may have underestimated the prevalence of CD slightly. Though 1% of our subjects were IgA deficient, it is unlikely that most of them had CD. A study of 126 IgA deficient children found the prevalence of anti TTG abtibodies was 21%, and prevalence od CD was 8.7% (26). A second study found that the prevalence of anti TTG-IgG was 9.8% among 174 blood donors with IgA deficiency (27). Thus, believe that excluding the IgA deficient individuals is unlikely to have influenced our overall findings. We collected tTG IgA from diagnosed JIA patients and controls, but we did not look into those with a diagnosis of CD with extraintestinal manifestations. Lastly, as it was a retrospective study, referral of all subjects to pediatric gastroenterology for complete evaluation of CD could not be performed.

**CONCLUSIONS:**

Using the largest JIA cohort to date to investigate prevalence of celiac antibodies, we show that the prevalence of positive tTG IgA was 1.9% and was not higher than among healthy controls. Furthermore, the prevalence of IgA deficiency was 1.2% like what has been reported. Together, our results did not demonstrate a higher prevalence of abnormal tTG IgA in JIA and thus our results do not support routine screening of celiac disease in JIA patients without symptoms of CD.

**LIST OF ABBREVIATIONS:**

| CD  JIA  tTG  DGP IgA/DGP IgG  EMA  HLA  NASPGHAN | Celiac Disease  Juvenile Idiopathic Arthritis  tissue transglutaminase  anti-deamidated gliadin peptide IgA/IgG anti-endomysium IgA  Human Leukocyte Antigen  North American Society for Pediatric Gastroenterology Hepatology and Nutrition |
| --- | --- |

**DECLARATIONS**

**ETHICS APPROVAL AND CONSENT TO PARTICIPATE** The study complied with the Declaration of Helsinki, was approved by ethic committees of Emory University and University of Utah, and written informed consent was obtained from the legally authorized representatives of the juvenile subjects, or the subject themselves. All subjects signed consents by IRB of Emory University School of Medicine or University of Utah.

**CONSENT FOR PUBLICATION** Not applicable

**AVAILABILITY OF DATA AND MATERIALS** The datasets generated and analyzed during the current study will be provided on request.

**COMPETING INTERESTS** Dr. Prahalad has served on a macrophage activation syndrome adjudication committee for Novartis, but this had no bearing on the work described in this manuscript. None of the other authors have any competing interests in the manuscript.

**FUNDING** Dr. Prahalad is supported in part by a grant from the Marcus Foundation Inc., Atlanta.

**AUTHORS CONTRIBUTIONS** ATK made substantial contributions to the study, including conceptualization/design, methodology, investigation, supervision/oversight, data curation, formal analysis, and resources. ATK wrote the first draft of the manuscript, supervised the analyses, reviewed, and edited the final manuscript. SP made substantial contributions to the study, including conceptualization/design, methodology, investigation, supervision/oversight, funding acquisition, data curation, formal analysis, and resources. SP wrote the first draft of the manuscript, supervised the analyses, reviewed the final manuscript. AH made substantial contributions to the study, including methodology, investigation, data curation, and review of the manuscript. JB made substantial contributions to the study including, conceptualization/design, methodology, funding acquisition, data curation, formal analyses, resources, and reviewed the manuscript. LP made substantial contributions to the study, including conceptualization/design, methodology, investigation, data curation. LHKC made substantial contributions to the study, including methodology, data curation, and review of the manuscript. SK made substantial contributions to the study, including methodology, investigation, supervision/oversight, and review the manuscript. KRS made substantial contributions to the study, including, methodology, investigation, supervision/oversight, and reviewed the manuscript. All authors read and approved the final manuscript.

**ACKNOWLEDGEMENTS** We gratefully acknowledge discussions with Pediatric Endocrinologist Dr. Andrew Muir, Pediatric Gastroenterologist Dr. Bess T Schoen, and Pediatric Rheumatologist Dr. Sheila Angeles-Han for critical inputs.

**TABLES and FIGURE**

**Table 1.** Characteristics of the subjects included in the study

**Table 2.** Studies of prevalence of tTG in cohorts of children with JIA

**Table 3.** Demographic features of cases and controls

**Table 4.** Phenotypes of the 15 children with high TTG IGA in our study

**Figure 1.** Serum IgA and IgA- anti tTG levels in the healthy controls and subjects with JIA

Table 1: Characteristics of the subjects included in the study

| JIA Category | Number | Mean age* (range*)+-SD* | Female  % | Positive TTG  N (%) | IGA deficiency  N(%) |
| --- | --- | --- | --- | --- | --- |
| Healthy Controls | 140 | 20.7 (3-30) +-4.2 | 54.3 | 5 (3.6) | 0 |
|  |  |  |  |  |  |
| All JIA | 808 | 10.9 (3-21) +-4.5 | 65.8 | 15 (1.9) | 9 (1.1) |
| Oligoarthritis, persistent | 237 | 9.2 (3-21) +-4.6 | 64.5 | 5 (2.1) | 2 (0.8) |
| Oligoarthritis, extended | 76 | 9.4 (3-18) +-4.6 | 77.3 | 0 | 2(2.6) |
| Polyarthritis, RF negative | 213 | 11.2 (3-21) +-4.3 | 76.5 | 4 (1.9) | 2 (0.9) |
| Polyarthritis, RF positive | 82 | 13.7 (4-18) +-3.6 | 85.3 | 4(5.0) | 2 (2.4) |
| Enthesitis related arthritis | 91 | 13.2 (3-21) +-3.3 | 34.0 | 1 (1.1) | 1 (1.1) |
| Systemic arthritis | 68 | 9.8 (3-19) +-4.4 | 42.6 | 1(1.5) | 0 |
| Psoriatic arthritis | 20 | 12.9 (9-17) +-2.7 | 35.0 | 0 | 0 |
| Undifferentiated arthritis | 21 | 11.1 (4-15) +-3.9 | 66.6 | 1 (1.5) | 0 |

*Years

Prevalence of IGA Def and TTG positivity were not significantly different between JIA cases and HC by Fisher’s Exact test. Prevalence of TTG positivity was also not significantly different between healthy controls and JIA categories.

Table 2: Studies of prevalence of tTG in cohorts of children with JIA

| Year | Country | Author | Controls  N | JIA  N | Biopsy confirmed | TTG Prevalence  controls | TTG Prevalence in JIA | Comments |
| --- | --- | --- | --- | --- | --- | --- | --- | --- |
| 2005 | Italy | Stagi | 158 | 151 | 10/10 | 0% | 6.7% | tTG IgA tested in 10/158 |
| 2008 | Italy | Alpigiani | 0 | 108 | 2/2 | - | 0.9% | tTG IgA = 1/108; IgA def = 0; |
| 2010 | Brazil | Koehne | 0 | 32 | ND | - | 0% | 11 EMA positive children were tested for tTG. All negative |
| 2012 | Egypt | Gheita | 30 | 42 | ND | 20% | 53% | tTG IgA jSPA (50%), JRA (53%), control (20%) |
| 2012 | USA | Stoll | 10 | 42 | 0/1 | 20% | 14% | tTG HC 2/10, JIA 3/31, ERA 3/11 |
| 2013 | Brazil | Robazzi | 40 | 53 | 1/1 | 0% | 1.9% | tTG IgA (N=1/53) |
| 2016 | Turkey | Moghtaderi | 0 | 53 | 0/1 | - | 1.8% | tTG IgA 1/53 (1.88%) |
| 2018 | Sweden | Oman | 0 | 216 | 3/4 | - | 2.8% | tTG IgA (3/213; 1.4%), IgA def (2/211); 3 prior CD |
| 2019 | Turkey | Sahin | 0 | 96 | ND | - | 0% | tTG IgA (0/96), IgA def (0/96) |
| 2023 | USA | Taneja | 140 | 808 | 2 | 3.6% | 1.9% |  |

N=Number, JIA: ND: not determined, TTG, Def: deficiency, JRA: , HC, CD, ERA: . jSPA: 9 prior studies have reported results of TTG antibody prevalence in JIA. Five of these had no healthy controls. Three of the studies reported the prevalence of IgA deficiency in their JIA cohorts.

Table 3: Demographic features of cases and controls

| **Category** | **Cases**  **(N=808)** | **TTG IgA**  **Positive (N)** | **IgA deficient**  **(N)** | **Controls**  **(N=140)** | **TTG IgA positive**  **(N)** |
| --- | --- | --- | --- | --- | --- |
| Sex |  |  |  |  |  |
| Female | 532 (65.8) | 9 | 6 | 65 (53.6) | 3 |
| Male | 276 (34.2) | 6 | 3 | 65 (46.4) | 2 |
| Race / Ethnicity |  |  |  |  |  |
| Hispanic n (%) | 44 (5.4) | 1 | 1 | 11 (7.9) | 0 |
| Non-Hispanic White n (%) | 636 (78.7) | 12 | 8 | 95 (67.9) | 5 |
| Blacks n (%) | 66 (8.1) | 0 | 0 | 9 (6.4) | 0 |
| Asian | 14 (1.7) | 0 | 0 | 13 (9.3) | 0 |
| Other/NR | 48 (5.9) | 2 | 0 | 12 (8.5) | 0 |

N: There were no differences in tTG positivity between cases and controls after stratification by sex or race/ethnicity.

Table 4: Phenotypes of the 15 children with high TTG IGA in our study

| ID1 | TTG_IGA | Gender | Race | Ethnicity | ILAR | collection age | Onset age | Comments |
| --- | --- | --- | --- | --- | --- | --- | --- | --- |
| 1 | 4 | F | W | NH | Oligo | 5 | 3.5 |  |
| 2 | 4 | F | W | NH | Poly RF N | 18 | 13 |  |
| 3 | 4 | F | W | NH | Poly RF N | 17 | 5 |  |
| 4 | 6 | F | W | NH | Oligo | 5 | 5 | TTG IGA negative on repeat testing |
| 5 | 6 | M | W | H | Poly RF P | 10 | 10 | Underwent EGD/C – negative for CD |
| 6 | 7 | M | W | NH | Oligo | 15 | 14 |  |
| 7 | 7 | M | W | NH | Systemic | 11 | 3 | Underwent EGD/C – negative for CD |
| 8 | 7 | M | W | H | Poly RF P | 10 | 2 |  |
| 9 | 12 | M | W | NH | ERA | 16 | 9 | Underwent EGD/C – negative for CD |
| 10 | 19 | F | W | NH | Poly RF N | 11 | 8 | TTG IGA negative on repeat testing |
| 11 | 30 | M | W | NH | Poly RF P | 15 | 2 | Diagnosed with CD endoscopically |
| 12 | 55 | F | W | NH | Poly RF P | 13 | 12 |  |
| 13 | 68 | F | W | NH | Oligo | 13 | 12 |  |
| 14 | 79 | F | W | NH | Poly RF N | 11 | 10 |  |
| 15 | >100 | F | W | NH | Oligo | 17 |  | Diagnosed with CD as adult. |
|  |  |  |  |  |  |  |  |  |

Abbreviations: TTG IGA: tissue transglutaminase IgA, F: female, M: male, W: white, NH: non-Hispanic, H: Hispanic, Oligo: Oligoarticular, Poly RF N: polyarticular RF negative, Poly RF positive: polyarticular RF positive, ERA: enthesitis-related JIA, EGD/C: endoscopy/colonoscopy, CD: celiac disease

Figure 1: Serum IgA and IgA- anti tTG levels in the healthy controls and subjects with JIA

AA
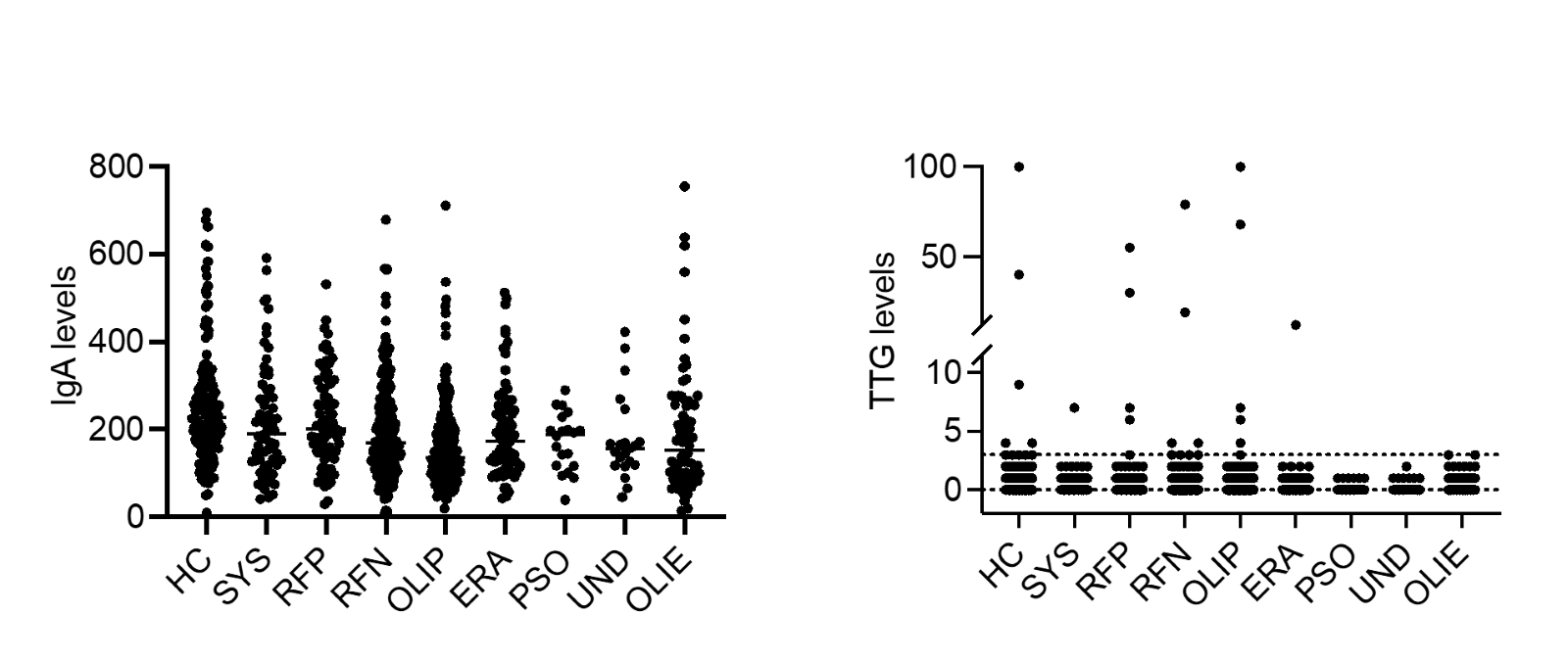


Abbreviations: HC: Healthy Control, SYS: Systemic JIA, RFP: RF Positive JIA, RFN: RF Negative JIA, OLIP: Oligoarticular JIA Persistent, ERA: Enthesitis-related JIA, PSO: Psoriatic JIA, UND: Undifferentiated JIA, OLIE, Oligoarticular JIA Extended

REFERENCES

1. Prahalad S, Zeft AS, Pimentel R, Clifford B, McNally B, Mineau GP, et al. Quantification of the familial contribution to juvenile idiopathic arthritis. Arthritis Rheum. 2010;62(8):2525-9.

2. Prahalad S, O'Brien E, Fraser AM, Kerber RA, Mineau GP, Pratt D, et al. Familial aggregation of juvenile idiopathic arthritis. Arthritis Rheum. 2004;50(12):4022-7.

3. Sandra Schenck JR, Martina Niewerth, Jens Klotsche, Kirsten Minden, Tobias Schwarz, Ivan Foeldvari, Gerd Horneff, Frank, Weller-Heinemann RWH, Angelika Thon. Comorbidity of Type 1 Diabetes Mellitus in Patients with Juvenile

Idiopathic Arthritis. The Journal of Pediatrics. 2017.

4. Prahalad S, Shear ES, Thompson SD, Giannini EH, Glass DN. Increased prevalence of familial autoimmunity in simplex and multiplex families with juvenile rheumatoid arthritis. Arthritis Rheum. 2002;46(7):1851-6.

5. De Maddi F, Pellegrini F, Raffaele CG, Tarantino G, Rigante D. Celiac disease and juvenile idiopathic arthritis: a still enigmatic crossover. Scandinavian journal of gastroenterology. 2013;48(4):511-2.

6. Alpigiani MG, Haupt R, Parodi S, Calcagno A, Poggi E, Lorini R. Coeliac disease in 108 patients with juvenile idiopathic arthritis: a 13-year follow-up study. Clin Exp Rheumatol. 2008;26(1):162.

7. Koehne Vde B, Bahia M, Lanna CC, Pinto MR, Bambirra EA, Cunha AS. Prevalence of serological markers for celiac disease (IgA and IgG class antigliadin antibodies and IgA class antiendomysium antibodies) in patients with autoimmune rheumatologic diseases in Belo Horizonte, MG, Brazil. Arquivos de gastroenterologia. 2010;47(3):250-6.

8. Moghtaderi M, Farjadian S, Aflaki E, Honar N, Alyasin S, Babaei M. Screening of patients with juvenile idiopathic arthritis and those with rheumatoid arthritis for celiac disease in southwestern Iran. The Turkish journal of gastroenterology : the official journal of Turkish Society of Gastroenterology. 2016;27(6):521-4.

9. Öman A, Hansson T, Carlsson M, Berntson L. Evaluation of screening for coeliac disease in children with juvenile idiopathic arthritis. Acta Paediatr. 2019;108(4):688-93.

10. Robazzi TC, Adan LF, Pimentel K, Guimaraes I, Magalhaes Filho J, Toralles MB, et al. Autoimmune endocrine disorders and coeliac disease in children and adolescents with juvenile idiopathic arthritis and rheumatic fever. Clin Exp Rheumatol. 2013;31(2):310-7.

11. Sahin Y, Sahin S, Barut K, Cokugras FC, Erkan T, Adrovic A, et al. Serological screening for coeliac disease in patients with juvenile idiopathic arthritis. Arab journal of gastroenterology : the official publication of the Pan-Arab Association of Gastroenterology. 2019;20(2):95-8.

12. Stagi S, Giani T, Simonini G, Falcini F. Thyroid function, autoimmune thyroiditis and coeliac disease in juvenile idiopathic arthritis. Rheumatology (Oxford). 2005;44(4):517-20.

13.

14. Ermarth A, Bryce M, Woodward S, Stoddard G, Book L, Jensen MK. Identification of Pediatric Patients With Celiac Disease Based on Serology and a Classification and Regression Tree Analysis. Clin Gastroenterol Hepatol. 2017;15(3):396-402.e2.

15. Hill ID, Dirks MH, Liptak GS, Colletti RB, Fasano A, Guandalini S, et al. Guideline for the diagnosis and treatment of celiac disease in children: recommendations of the North American Society for Pediatric Gastroenterology, Hepatology and Nutrition. Journal of pediatric gastroenterology and nutrition. 2005;40(1):1-19.

16.

17. Choung RS, Ditah IC, Nadeau AM, Rubio-Tapia A, Marietta EV, Brantner TL, et al. Trends and racial/ethnic disparities in gluten-sensitive problems in the United States: findings from the National Health and Nutrition Examination Surveys from 1988 to 2012. Am J Gastroenterol. 2015;110(3):455-61.

18. Fasano A, Berti I, Gerarduzzi T, Not T, Colletti RB, Drago S, et al. Prevalence of celiac disease in at-risk and not-at-risk groups in the United States: a large multicenter study. Arch Intern Med. 2003;163(3):286-92.

19. Pohjankoski H, Kautiainen H, Kotaniemi K, Korppi M, Savolainen A. Autoimmune diseases in children with juvenile idiopathic arthritis. Scand J Rheumatol. 2010;39(5):435-6.

20. Atteno M, Costa L, Cozzolino A, Tortora R, Caso F, Del Puente A, et al. The enthesopathy of celiac patients: effects of gluten-free diet. Clin Rheumatol. 2014;33(4):537-41.

21. 5.

REFERENCES

1. Prahalad S, Zeft AS, Pimentel R, Clifford B, McNally B, Mineau GP, et al. Quantification of the familial contribution to juvenile idiopathic arthritis. Arthritis Rheum. 2010;62(8):2525-9.

2. Prahalad S, O'Brien E, Fraser AM, Kerber RA, Mineau GP, Pratt D, et al. Familial aggregation of juvenile idiopathic arthritis. Arthritis Rheum. 2004;50(12):4022-7.

3. Sandra Schenck JR, Martina Niewerth, Jens Klotsche, Kirsten Minden, Tobias Schwarz, Ivan Foeldvari, Gerd Horneff, Frank, Weller-Heinemann RWH, Angelika Thon. Comorbidity of Type 1 Diabetes Mellitus in Patients with Juvenile

Idiopathic Arthritis. The Journal of Pediatrics. 2017.

4. Prahalad S, Shear ES, Thompson SD, Giannini EH, Glass DN. Increased prevalence of familial autoimmunity in simplex and multiplex families with juvenile rheumatoid arthritis. Arthritis Rheum. 2002;46(7):1851-6.

5. De Maddi F, Pellegrini F, Raffaele CG, Tarantino G, Rigante D. Celiac disease and juvenile idiopathic arthritis: a still enigmatic crossover. Scandinavian journal of gastroenterology. 2013;48(4):511-2.

6. Alpigiani MG, Haupt R, Parodi S, Calcagno A, Poggi E, Lorini R. Coeliac disease in 108 patients with juvenile idiopathic arthritis: a 13-year follow-up study. Clin Exp Rheumatol. 2008;26(1):162.

7. Koehne Vde B, Bahia M, Lanna CC, Pinto MR, Bambirra EA, Cunha AS. Prevalence of serological markers for celiac disease (IgA and IgG class antigliadin antibodies and IgA class antiendomysium antibodies) in patients with autoimmune rheumatologic diseases in Belo Horizonte, MG, Brazil. Arquivos de gastroenterologia. 2010;47(3):250-6.

8. Moghtaderi M, Farjadian S, Aflaki E, Honar N, Alyasin S, Babaei M. Screening of patients with juvenile idiopathic arthritis and those with rheumatoid arthritis for celiac disease in southwestern Iran. The Turkish journal of gastroenterology : the official journal of Turkish Society of Gastroenterology. 2016;27(6):521-4.

9. Öman A, Hansson T, Carlsson M, Berntson L. Evaluation of screening for coeliac disease in children with juvenile idiopathic arthritis. Acta Paediatr. 2019;108(4):688-93.

10. Robazzi TC, Adan LF, Pimentel K, Guimaraes I, Magalhaes Filho J, Toralles MB, et al. Autoimmune endocrine disorders and coeliac disease in children and adolescents with juvenile idiopathic arthritis and rheumatic fever. Clin Exp Rheumatol. 2013;31(2):310-7.

11. Sahin Y, Sahin S, Barut K, Cokugras FC, Erkan T, Adrovic A, et al. Serological screening for coeliac disease in patients with juvenile idiopathic arthritis. Arab journal of gastroenterology : the official publication of the Pan-Arab Association of Gastroenterology. 2019;20(2):95-8.

12. Stagi S, Giani T, Simonini G, Falcini F. Thyroid function, autoimmune thyroiditis and coeliac disease in juvenile idiopathic arthritis. Rheumatology (Oxford). 2005;44(4):517-20.

13. Ermarth A, Bryce M, Woodward S, Stoddard G, Book L, Jensen MK. Identification of Pediatric Patients With Celiac Disease Based on Serology and a Classification and Regression Tree Analysis. Clin Gastroenterol Hepatol. 2017;15(3):396-402.e2.

14. Hill ID, Dirks MH, Liptak GS, Colletti RB, Fasano A, Guandalini S, et al. Guideline for the diagnosis and treatment of celiac disease in children: recommendations of the North American Society for Pediatric Gastroenterology, Hepatology and Nutrition. Journal of pediatric gastroenterology and nutrition. 2005;40(1):1-19.

15. Choung RS, Ditah IC, Nadeau AM, Rubio-Tapia A, Marietta EV, Brantner TL, et al. Trends and racial/ethnic disparities in gluten-sensitive problems in the United States: findings from the National Health and Nutrition Examination Surveys from 1988 to 2012. Am J Gastroenterol. 2015;110(3):455-61.

16. Fasano A, Berti I, Gerarduzzi T, Not T, Colletti RB, Drago S, et al. Prevalence of celiac disease in at-risk and not-at-risk groups in the United States: a large multicenter study. Arch Intern Med. 2003;163(3):286-92.

17. Pohjankoski H, Kautiainen H, Kotaniemi K, Korppi M, Savolainen A. Autoimmune diseases in children with juvenile idiopathic arthritis. Scand J Rheumatol. 2010;39(5):435-6.

18. Atteno M, Costa L, Cozzolino A, Tortora R, Caso F, Del Puente A, et al. The enthesopathy of celiac patients: effects of gluten-free diet. Clin Rheumatol. 2014;33(4):537-41.

19. Skrabl-Baumgartner A, Christine Hauer A, Erwa W, Jahnel J. HLA genotyping as first-line screening tool for coeliac disease in children with juvenile idiopathic arthritis. Archives of disease in childhood. 2017;102(7):607-11.

20. Petty RE, Southwood TR, Manners P, Baum J, Glass DN, Goldenberg J, et al. International League of Associations for Rheumatology classification of juvenile idiopathic arthritis: second revision, Edmonton, 2001. J Rheumatol. 2004;31(2):390-2.

21. Prahalad S, Hansen S, Whiting A, Guthery SL, Clifford B, McNally B, et al. Variants in TNFAIP3, STAT4, and C12orf30 loci associated with multiple autoimmune diseases are also associated with juvenile idiopathic arthritis. Arthritis Rheum. 2009;60(7):2124-30.

22. Schyum AC, Rumessen JJ. Serological testing for celiac disease in adults. United European gastroenterology journal. 2013;1(5):319-25.

23. Ciccocioppo R, Kruzliak P, Cangemi GC, Pohanka M, Betti E, Lauret E, et al. The Spectrum of Differences between Childhood and Adulthood Celiac Disease. Nutrients. 2015;7(10):8733-51.

24. Gheita TA, Fawzy SM, Nour El-Din AM, Gomaa HE. Asymptomatic celiac sprue in juvenile rheumatic diseases children. International journal of rheumatic diseases. 2012;15(2):220-6.

25. Stoll ML, Patel AS, Christadoss ML, Punaro M, Olsen NJ. IgA transglutaminase levels in children with Juvenile Idiopathic Arthritis. Annals of paediatric rheumatology. 2012;1(1):31-5.

26. Lenhardt A, Plebani A, Marchetti F, Gerarduzzi T, Not T, Meini A, et al. Role of human-tissue transglutaminase IgG and anti-gliadin IgG antibodies in the diagnosis of coeliac disease in patients with selective immunoglobulin A deficiency. Dig Liver Dis. 2004;36(11):730-4.

27. Korponay-Szabó IR, Dahlbom I, Laurila K, Koskinen S, Woolley N, Partanen J, et al. Elevation of IgG antibodies against tissue transglutaminase as a diagnostic tool for coeliac disease in selective IgA deficiency. Gut. 2003;52(11):1567-71.
