# Supplementary material for: Prevalence of tissue transglutaminase antibodies and IgA deficiency are not increased in juvenile idiopathic arthritis: a case-control study
Source: Pediatr Rheumatol Online J. 2023 Oct 5;21:110. doi: 10.1186/s12969-023-00890-z (PMC10557180; doi:10.1186/s12969-023-00890-z)
Supplement: Supplementary file 2 — Supplementary Material 2 [file 12969_2023_890_MOESM2_ESM.docx]

August 15, 2023

Prof Alberto Martini and Prof Charles Spencer

Editors

Pediatric Rheumatology BMC

Dear Professors Alberto Martini and Charles Spencer,

Thank you for giving us the opportunity to re-submit the article entitled “*Prevalence of tissue Transglutaminase Antibodies and IgA deficiency are not increased in Juvenile Idiopathic Arthritis: A case control study*” to be considered for publication in *Pediatric Rheumatology BMC.* We have responded to reviewers' comments.

We appreciate the opportunity given to re-submit. We have adhered to the guidelines provided in the instructions to the authors for a Research Article. Also, thank you for accepting the request for deadline extension. *Manuscript ID: PROJ-D-23-0082.*

We look forward to your decision. Please do not hesitate to contact me if you need any additional information.

Sincerely yours,

Angela Taneja Kohli, MD

Assistant Professor of Pediatrics

Division of Pediatric Rheumatology

angela.taneja@emory.edu
